# Evaluation of Different Tools of Precision Finishing for Sun Gear with Inner-Outer Tooth Shapes

**DOI:** 10.3390/ma15228081

**Published:** 2022-11-15

**Authors:** Qiang Liang, Xianming Zhang, Yunqi Liu, Jie Zhou, Zuofa Liu

**Affiliations:** 1 College of Mechanical Engineering, Chongqing Technology and Business University, Chongqing 400067, China; 2 Engineering Research Center for Waste Oil Recovery Technology and Equipment of Ministry of Education, Chongqing Technology and Business University, Chongqing 400067, China; 3 Chongqing Key Laboratory of Advanced Mold Intelligent Manufacturing, College of Materials Science and Engineering, Chongqing University, Chongqing 400044, China

**Keywords:** sun gear, precision finishing, tooth accuracy, inner-outer tooth shapes, finite element prediction

## Abstract

Aiming at the technical difficulty of poor teeth accuracy of the extruded sun gears, an innovative precision finishing approach was developed as the subsequent process of producing high-quality sun gears, and three finishing tools were designed, namely mandrel with gap, mandrel without gap and interference mandrel. A finite element prediction method was proposed by using the post-processing toolbox of DEFORM software to study the distribution laws of outer and inner teeth deviations of the reshaped sun gear. Moreover, the effects of various precision finishing tools on the formability of sun gear (such as tooth deformation, tool stress and tooth accuracy) were investigated. The results show that the tooth accuracy class of the reshaped outer teeth in profile and helix were seventh and eighth, respectively, and the total *M*-value error of the inner teeth was decreased to 72.3 μm and the reduction ratio was 73.8% by adopting the interference mandrel, which indicates that the finishing effect of the interference mandrel was better than that of mandrels for both with gap and without gap, and the forming accuracy of the finished teeth could be considerably improved. Therefore, the interference mandrel can be recommended as the optimum finishing tool in the manufacture of precision sun gears. The simulated results were in good agreement with experimental ones within a maximum error of 14.4%, which proves the practicability of the sizing approach and the dependability of the finite element prediction method.

## 1. Introduction

As a classical power transmission part, sun gear with inner and outer teeth has been widely used in automobile driving systems due to its reliable operation, high transmission efficiency and wide applicability [1,2]. As gear manufacturing is a mass production process, precision forging gears should not only ensure continuous and efficient production, but also ensure a reliable die and mold [3,4]. With the development of cold extrusion technology, this net near forming method is more and more adopted in the manufacture of gears. The cold extruded gears have smooth surfaces, continuous metal streamlines and high bearing capacity [5,6]. Therefore, cold extrusion technology has gradually become the main gear processing method. Silveira and Schaeffer [7] evaluated the effects of different die prestress on the dimensional accuracy of extruded gear and die stress and compared to the application effects of the two kinds of shrinkage rings in practical production. Tiernan et al. [8] examined the effects of deformation rate and tool shape on force in the forming process of cold extruded aluminum alloy parts using numerical simulation and experiment methods and analyzed the correlation between simulated and experimental results.

While cold extrusion gear technology has remarkable superiorities, the dimensional accuracy of the cold extruded teeth is inferior, which cannot satisfy the demand of precision-forged gear and can only be used as a pre-forging part [9,10]. Hence, the precision sizing of cold extruded teeth is very valuable. Stone et al. [11] compared the influences of one or two cold finishing processes on the surface morphology and dimensional accuracy of thick-wall cylindrical parts. The results show that the finishing process can significantly enhance the teethed quality. Li et al. [12] studied the effect of the subsequent finishing process on the forming quality of hot forged helical gears. They found that the reshaping process can remarkably improve the surface quality of gears, but over finishing will reduce the surface roughness. The compound process of cold or hot forging and cold finishing was developed by Behrens and Doege [13]. Aiming at the problem of die elastic deformation during the cold sizing process, the strategies of finite element simulation to predict tooth deviation and reverse compensation die were proposed. Pfefferkorn et al. [14] simulated the influence of elastic deformation of die and workpiece on single tooth deviation, which was verified by experiments. The results demonstrated that this method could effectively predict the dimensional deviation, and the tooth surface roughness could be improved. Liu et al. [15] simulated and analyzed the whole process of warm forging-cold extrusion-cold finishing of large module spur gear by employing the finite element (FE) method and investigated the influence of hollow blank size on material flow and forming load.

Until now, the prediction of tooth deviation is one of the key technical difficulties in the precision finishing of gears. This is due to the material flow of the gear being extremely complicated during the extrusion process, which makes the tooth deviations uncontrollable. Ou and Balendra [16] presented the strategy of simulating and predicting tooth deviation and die elastic compensation to manufacture high-precision gear products. Lee et al. [17] developed a novel finite element modeling approach to predict the dimensional deviation of cold-forged gears. The predicted results were in good agreement with the experimental ones. Zhuang et al. [18] developed a new FE prediction method to realize the visualization of tooth size change of hot-forged bevel gears. The experimental results show that the prediction method was feasible and reliable. Kang et al. [19] considered elastic deformation in the manufacturing process of gear die, resulting in the forming accuracy of the gear being significantly improved. Additionally, the feasibility of the die manufacturing method was proved by simulation prediction and process experiment. Qin and Balendra [20] predicted the geometric dimension changes of formed gears under die loading and unloading using numerical simulation. They found that the elastic deformation of the loaded die had a great influence on the dimensional deviation of the extrusion gears, and the deviation would be maintained after the die was unloaded.

In summary, remarkable achievements have been made in the precision forming of outer teeth or inner teeth, but the report concerning the tooth deviations of cold extruded sun gears with outer and inner teeth is rare. In this work, an innovative precision sizing approach was developed as the subsequent process of producing high-quality sun gears, and three finishing tools were designed, namely mandrel with gap, mandrel without gap and interference mandrel. The profile curve of spline mandrel without gap is the same as that of the objective inner tooth; the dimensional profile of mandrel with gap is 0.1 mm smaller than that of the objective inner tooth so that there is a clearance of 0.1 mm between the spline mandrel and the billet; the dimensional profile of interference mandrel is 0.1 mm larger than that of the objective inner tooth. Then, a FE prediction method was proposed to investigate the distribution laws of outer and inner teeth deviations of the finished sun gear. To prove the practicability of the sizing approach and the dependability of the finite element prediction method, experiments of different finishing tools were conducted. Finally, the effects of various precision finishing tools on the formability of sun gear (such as tooth deformation, tool stress and tooth accuracy) were investigated to determine the optimum finishing tool in the manufacture of precision sun gears.

## 2. Precision Finishing Approach

### 2.1. Cold Extruded Sun Gear

**Figure 1:**
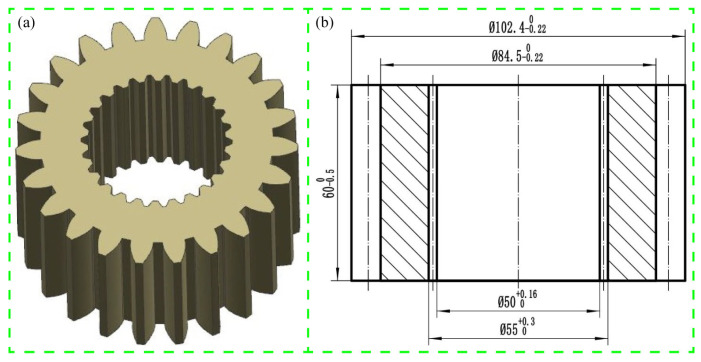
Extrusion drawing of sun gear: (**a**) 3D structure; (**b**) 2D drawing.

The precision sun gear part has been commonly employed in automobile power conveying systems due to it having internal and external tooth shapes. Figure 1 presents the extrusion drawing of the sun gear. Based on the structural characteristics of the sun gear, a two-step extrusion process was proposed, where the inner teeth extruded first, and the outer teeth then extruded.

According to ISO 1328, the important index of internal teeth accuracy is the total *M* value error (*F_M_*), and the evaluation index of the outer teeth accuracy is the total profile error (*F_α_*) and total helix error (*F_β_*). The dimension accuracy of inner and outer teeth obtained by the cold extrusion process was detected using the gear detection center (Model 3906 gear detection center made by Genertec Harbin Measuring & Cutting Tool Co., Ltd., Harbin, China ), as given in Table 1. It can be seen that the tooth accuracy class of profile and lead of the outer teeth were ninth and tenth, respectively, and both were unable to achieve the product requirements of precision gears (accuracy class eighth). For the inner teeth, the total *M*-value error was 276.4 μm, which was far greater than the 100 μm required by the precision gears. Therefore, the precision sizing of cold extruded teeth is very valuable.

### 2.2. Precision Finishing Method Design

**Figure 2:**
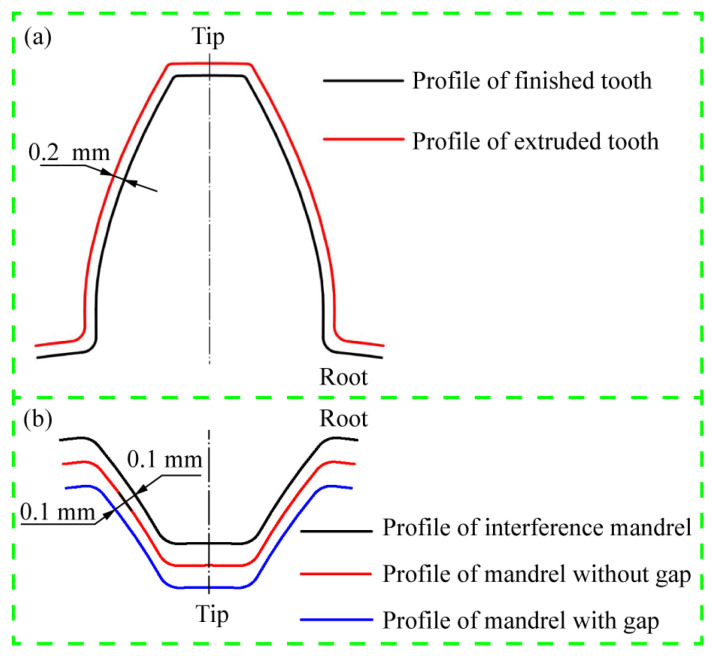
Schematic view of different finishing tools for sun gear: (**a**) Outer teeth and (**b**) inner teeth.

**Figure 3:**
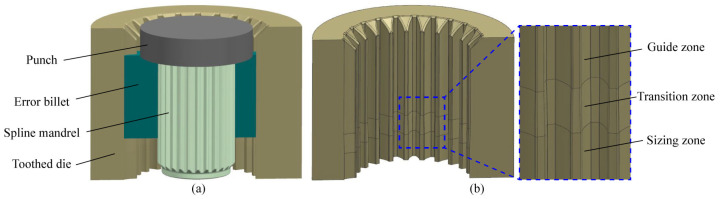
Precision finishing process of inner-outer teeth [21]: (**a**) Assembly diagram and (**b**) toothed die structure.

To enhance the dimensional precision of the cold-formed teeth, an innovative finishing approach to the simultaneous sizing of internal and external teeth was designed. The principle was to use the interference shaping constraint of the external tooth die to promote the radial flow of metal, leading to improvement of the fit degree between the spline mandrel and the internal tooth. The schematic view of the shaping outer teeth is shown in Figure 2a. The entire tooth surface of the external tooth will be finished by 0.2 mm. While the internal teeth will be finished by three kinds of spline mandrels, which are mandrel with gap, mandrel without gap and interference mandrel, as shown in Figure 2b. The profile curve of spline mandrel without gap is the same as that of the objective inner tooth; the dimensional profile of mandrel with gap is 0.1 mm smaller than that of the objective inner tooth so that there is a clearance of 0.1 mm between the spline mandrel and the billet; the dimensional profile of the interference mandrel is 0.1 mm larger than that of the objective inner tooth, resulting in the fit degree between the spline mandrel and the internal tooth can be enhanced. In the finishing process, the inner tooth was traversed first by the spline mandrel, and then the external teeth were shaped under the action of the press, as illustrated in Figure 3.

## 3. Simulation and Experiment Preparation

### 3.1. FE Modeling and Prediction Strategy

**Figure 4:**
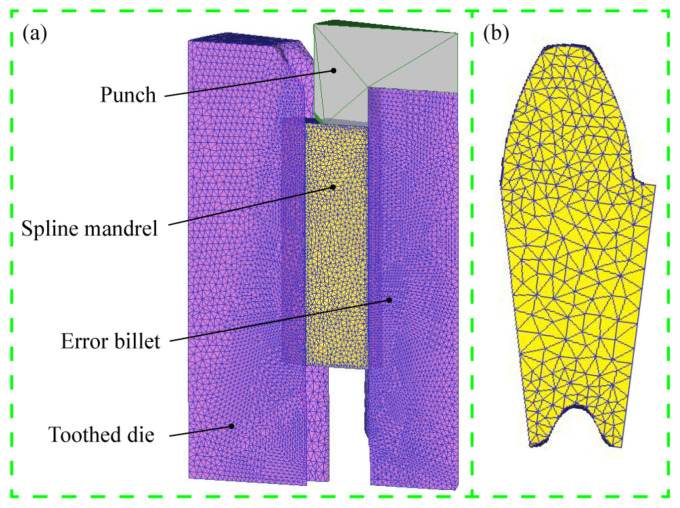
Numerical simulation model of precision finishing: (**a**) Assembly bodies and (**b**) cross section of billet.

To study the effects of various precision finishing tools on the formability of sun gear, such as tooth deformation, tool stress and tooth accuracy, an FE model for simulation and prediction was established, as shown in Figure 4. In References [13,22,23], the ideal workpiece model without error was employed in the FE simulation of finishing spur gears, and the gear accuracy of ideal billet was naught class, which was quite different from the actual gear accuracy of the tenth class after cold extrusion. To be more similar to the actual production, the error billet model was established based on the measured *M* value and tooth thickness for the numerical simulation of different finishing tools. A 1/26 section model (single tooth) was applied for FE simulation to reduce the calculation time. Considering the elastic deformation in the finishing process, the error billet, spline mandrel and toothed die were defined as elastic-plastic bodies, and the other tools were considered as rigid bodies. To improve the simulation accuracy, the toothed regions of workpiece and tools were locally refined, and the proportion was 0.1. Table 2 presents the key parameters for the FE simulation. The material of the error billet is SCM 420, and its chemical composition is given in Table 3.

**Figure 5:**
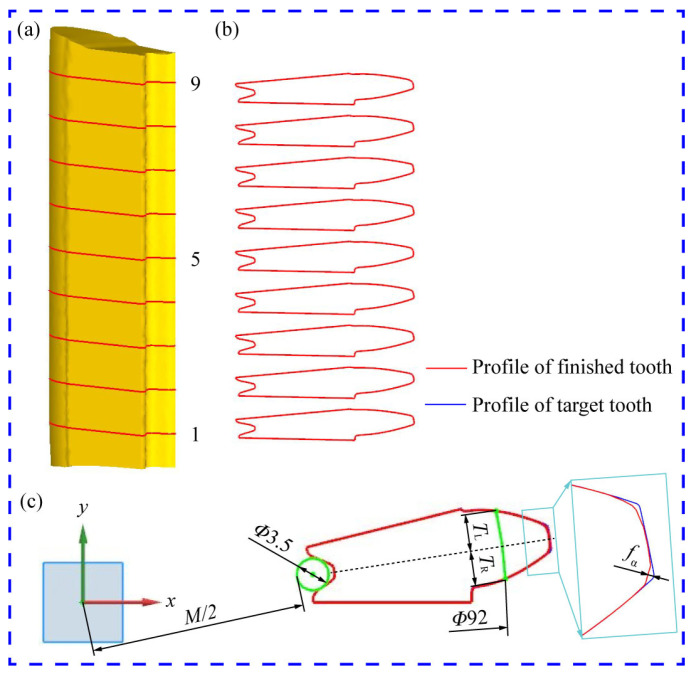
Prediction approach of tooth deviations: (**a**) Finished teeth; (**b**) extracted tooth profiles; and (**c**) measurment view.

Most research on the dimensional deviation of precision gears primarily focused on the total deviations of tooth helix and profile [18,24]. However, due to the complex structure of the sun gear and the complicated metal flow in the sizing process, the dimensional deviation of the teeth is difficult to control. To investigate the distribution law of dimensional error of finished teeth, an accurate finite element prediction method was developed in this paper, as presented in Figure 5. Firstly, the reshaped teeth were divided into nine sections along the axis, and nine tooth profile curves can be extracted using the post-processing toolbox of DEFORM-3D software. Then, the deviations of the extracted tooth profiles were measured using UG modeling software. The total *M*-value error of the internal teeth was assumed to be the maximum *M*-value difference between the reshaped and objective teeth, the total tooth profile deviation of the outer teeth was assumed to be the maximum value of the single tooth profile deviation (*f_α_*), and the total helix deviation was assumed to be the maximum difference between the left (*T*_L_) and right (*T*_R_) tooth thickness of the reshaped and target teeth. The detailed description of the prediction strategy can be seen in the previous research results obtained by our team [25].

### 3.2. Experiment Preparation

**Figure 6:**
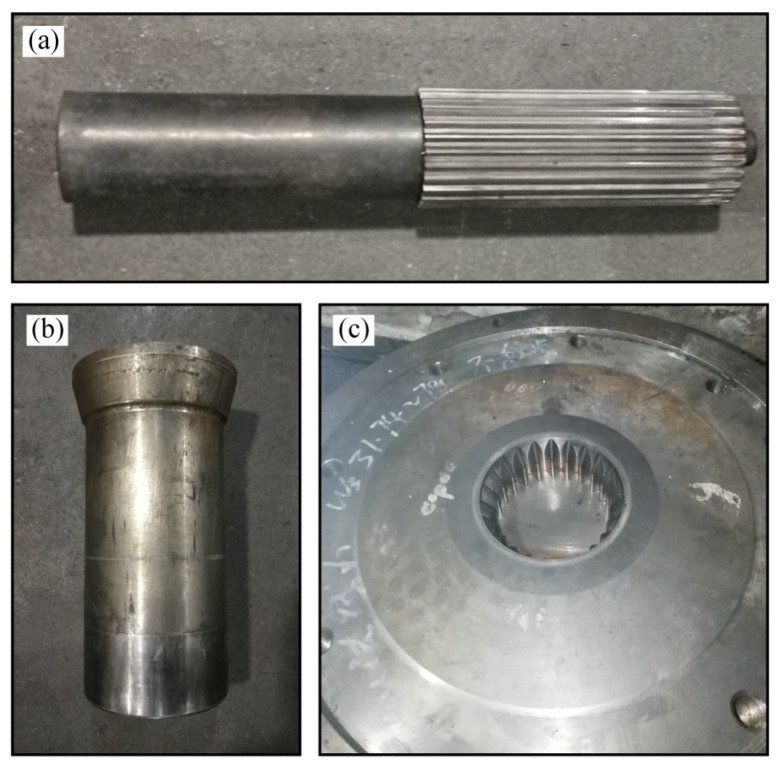
Experimental tools: (**a**) Mandrel; (**b**) punch; and (**c**) die.

Experiments of different finishing tools were carried out on a 4000 kN hydraulic press (YJ32-400T four column hydraulic press made by Chongqing Jiang Dong Machinery Co., Ltd., Chongqing, China) to prove the feasibility of the reshaping approach and prediction strategy. Figure 6 shows the experimental tools in the precise sizing process. The materials of tools were SKD11. To save on the experimental cost, the interference mandrel was produced first by the wire electrical discharge machine for the precision reshaping experiment. When the experiment in interference mandrel was complete, the dimensional profile of the toothed mandrel was machined by 0.1 mm to obtain the spline mandrel without gap, then the finishing experiment was conducted. Finally, the tooth profile of the mandrel without gap was processed by 0.1 mm to carry out the precision finishing experiment in the mandrel with gap. Moreover, three finished sun gears obtained by different reshaping tools were randomly selected to inspect the dimensional deviations of the outer and inner teeth. The total deviation of tooth profile, helix and *M* value was measured by selecting four approximately quartered teeth of the finished sun gears along the circumference of the gear. In addition, each measurement was carried out three times to obtain an accurate value.

## 4. Results and Discussion

### 4.1. Tooth Deformation

**Figure 7:**
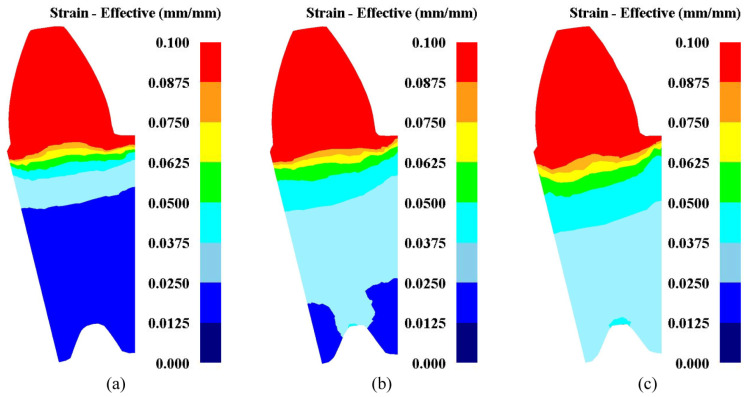
Effective strain of reshaped teeth at various finishing tools: (**a**) Mandrel with gap; (**b**) mandrel without gap; and (**c**) interference mandrel.

The effective strain distributions of deformation cross-sections with different reshaping tools are displayed in Figure 7. The deformation region of the outer tooth was the whole profile, but the internal spline was different because of different spline mandrels for the precision finishing operation. When the spline mandrel was with gap, rare plastic deformation was observed in the internal spline, as shown in Figure 7a; when the spline mandrel without gap was adopted, the billet would be restrained by the toothed tools, and the deformation zone of the inner tooth was mainly concentrated on the tooth tip, as displayed in Figure 7b; when the interference mandrel was applied, the billet fitted closely to the spline mandrel, leading to the deformation region of internal spline was the whole tooth surface, as denoted in Figure 7c. This is basically consistent with the previous results that the distribution law of the effect variation of using three reshaping methods, such as full tooth sizing, tooth root sizing and sizing only tooth face [26]. To sum up, when the interference mandrel is used for the precision finishing process, the deformation zones of the finished sun gear are uniformly distributed and the entire profiles of internal–external teeth will undergo plastic deformation, so the finishing effect of the interference mandrel is better than the spline mandrel with gap and without gap.

### 4.2. Tool Stress

**Figure 8:**
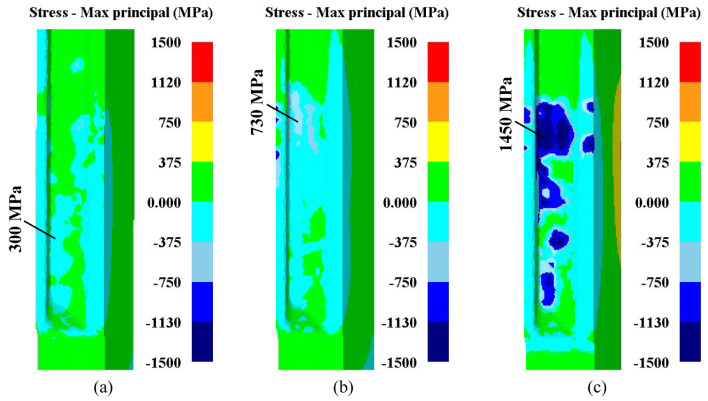
Max principal stress distributions of different spline mandrels: (**a**) Mandrel with gap; (**b**) mandrel without gap; and (**c**) interference mandrel.

In the precision finishing process, the tools (spline mandrel and toothed die) are subjected to considerable stresses exerted by the billet. It is well-known that smaller compressive stress on the tool surfaces means less plastic deformation and a greater operation time of tools. The max principal stress distributions of spline mandrels illustrated in Figure 8 are the FE simulation results in different finishing tools, where the negative value is compressive stress, and the positive value is tensile stress. The tooth tips of three kinds of spline mandrels are both endured to remarkable compressive stress during the precision finishing operations, which is the main reason for the plastic deformation of spline mandrels. When the spline mandrel was with gap, due to the clearance between the billet and mandrel, the compressive stress on the mandrel was the smallest, at about 300 MPa, as shown in Figure 8a. When the spline mandrel without gap was used, as illustrated in Figure 8b, the billet would be constrained by the spline mandrel, the compressive stress of the mandrel was primarily in the boundary of 0~375 MPa and the max value at the tooth tip was 730 MPa. When the interference mandrel was adopted, the maximum compressive stress at the tooth tip of the spline mandrel was 1450 MPa because the billet fitted well to the mandrel, as denoted in Figure 8c.

### 4.3. Tooth Accuracy

**Figure 9:**
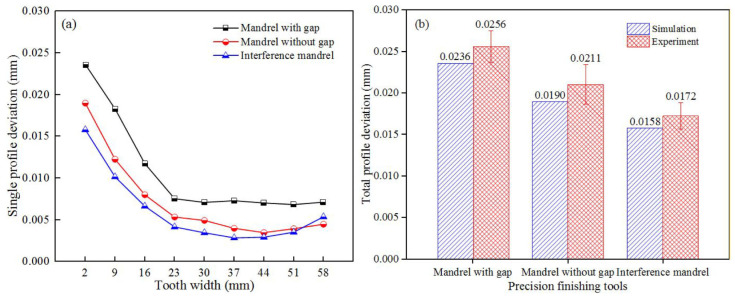
Profile deviations of external gear at different finishing tools: (**a**) Single and (**b**) total.

The single profile deviation distributions of the outer tooth at various finishing tools are illustrated in Figure 9a. It can be observed that the tooth profile errors initially diminished sharply and then tended to become stable gradually with the rise in tooth width. Moreover, the errors of reshaped teeth obtained by adopting interference mandrel were smaller than those obtained by using mandrels both with gap and without gap. This is due to the interference mandrel has a better constraint on the billet, which makes the finishing effect of the external gear preferable.

The total profile deviations of each experimental sun gear formed by different finishing tools are displayed in Figure 9b. The total profile deviation obtained using the interference mandrel was 0.0172 mm, being 32.8% and 18.5% lower than that using mandrels with gap and without gap, respectively. Additionally, it can be observed that the experimental results exceeded the numerically simulated ones. The dimensional differences between the reshaped teeth inspection and finite element prediction could be primarily attributed to the thermal deformation of the billet, which was not considered in this study.

**Figure 10:**
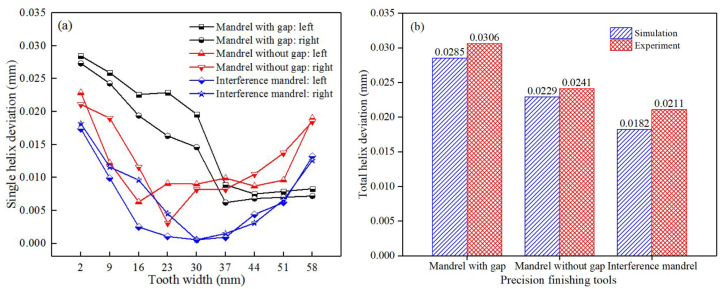
Helix deviations of external gear at different finishing tools: (**a**) Single and (**b**) total.

Figure 10 shows the helix errors of the outer teeth obtained from FE simulations and experiments. When the mandrel was with a gap, the single helix errors decreased remarkably and tended to be invariant with the increase in tooth width. Additionally, a max deviation of 0.0285 mm was observed at the end of small tooth width. When the spline mandrel without gap was adopted, as rising tooth width, the deviations diminished significantly at first, then kept stable and finally increased gradually. Moreover, the total helix deviations obtained from experiment and simulation were 0.0241 and 0.0229 mm respectively. When the interference mandrel was applied, the single helix errors decreased gradually and then rose remarkably with the rise in tooth width. Further, the total helix deviation of 0.0182 mm was observed at the end of small tooth width, being 36.1% and 20.5% lower than that using mandrels with gap and without gap, respectively. It can likewise be seen that the total helix deviations obtained by experiments were larger than that obtained by numerical simulations.

**Figure 11:**
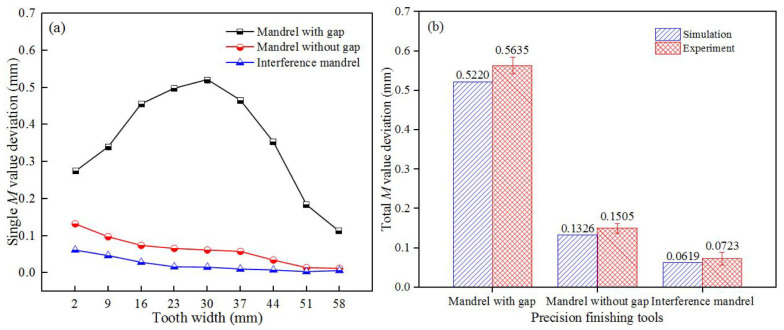
*M* value deviations of the inner tooth at various finishing tools: (**a**) Single and (**b**) total.

The *M*-value errors of inner tooth gained by both simulations and experiments are shown in Figure 11. As the rising tooth width, single *M*-value deviations in mandrel with gap grown firstly and then decreased remarkably, and the largest value of 0.5635 mm occurred on the middle region of the formed tooth. This may be explained that, during the process of precision finishing, due to the clearance between the billet and spline mandrel, materials will flow to the inner layer under the push of external gear reshaping, leading to the tiny upsetting phenomenon of big in the middle region and small at both ends. With the enlargement in tooth width, the single *M* value deviations obtained using mandrel without gap and interference mandrel reduced gradually, and the experimental results of the total *M* value errors were 0.1505 and 0.0723 mm, respectively. This finding shows that the constraint effect of the spline mandrel can effectively ensure the reshaping quality of the internal spline, which is consistent with the above analysis of the tools.

**Figure 12:**
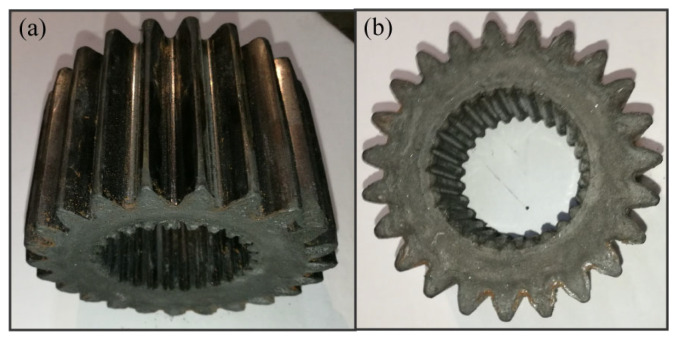
Finished sun gear: (**a**) Outer tooth shape and (**b**) inner tooth shape.

From the above analysis, it can be concluded that, compared to the spline mandrels, both with gap and without gap, the interference mandrel has a considerable reshaping effect for internal–external teeth due to the constraint function on the billet. Figure 12 presents the reshaped sun gear using the interference mandrel. It can be observed that the inner and outer teeth were filled, and the tooth surface was smooth without obvious defects, demonstrating the effectiveness of the precise finishing approach. The accuracy class of the precision finished teeth using the interference mandrel is listed in Table 4. It can be observed that the tooth accuracy class of the reshaped outer teeth in the profile and helix were seventh and eighth, respectively, and the total *M*-value error of the inner teeth was decreased to 72.3 μm, indicating that the dimensional accuracy of the cold extruded teeth can be considerably enhanced by the precise finishing operation. Therefore, the interference mandrel can be recommended as the optimum finishing tool in the manufacture of precise sun gears.

## 5. Conclusions

In this work, an innovative precision finishing approach was developed and three finishing tools were designed. A FE prediction method was proposed to investigate the distribution laws of outer and inner teeth deviations of the finished sun gear. The effects of various precision finishing tools on the formability of sun gear (such as tooth deformation, tool stress and tooth accuracy) were investigated to determine the optimum finishing tool in the manufacture of precise sun gears. The most important results of this study include:

(1) When the interference mandrel is used for the precision finishing process, the entire profiles of internal–external teeth will occur plastic deformation, and the finishing effect is better than mandrels both with gap and without gap. Therefore, the interference mandrel is recommended as the optimal reshaping tool to improve the tooth accuracy of cold extruded sun gear.
(2) While the maximum compressive stress of the tools with the interference mandrel is larger than mandrels both with gap and without gap, the interference mandrel has a better restraint function on the billet, which indicates that this finishing tool can significantly improve the reshaping effect of internal–external gears.
(3) The tooth accuracy class of the reshaped outer teeth in profile and helix were seventh and eighth, respectively, and the total *M*-value error of the inner teeth was decreased to 72.3 μm, indicating that the dimensional accuracy of the cold extruded teeth can be considerably improved by the precise finishing operation. The simulation results of dimensional errors were consistent with the experimental results, which verifies the dependability of the finite element prediction method.

In this study, the deformation temperature of the precision finishing process was assumed at room temperature and as a constant value in the FE simulation, which made the forming accuracy of the inner and outer teeth of the sun gear have some errors with the experimental gears. In actual production, the temperature of the billet and tools will rise remarkably due to the heat is generated during the forming process, which directly affects the tooth accuracy of the reshaped gears. In the following research, the effect of forming temperature on gear accuracy will be investigated.

## Figures and Tables

**Table 1:** Accuracy class of cold extruded teeth.

Deviations	Values	Accuracy Class (ISO 1328)
*F_M_*	276.4 μm	100 μm
*F_α_*	30.2 μm	9 class
*F_β_*	55.9 μm	10 class

**Table 2:** Parameters for the FE simulation.

Items	Values
Error billet material	SCM 420
Finishing tools material	SKD11
Error billet mesh number	100,000
Finishing tools mesh number	100,000
Mesh refined proportion of billet and tools	0.1
Mesh size of billet and tools	1
Finishing temperature (°C)	20
Pressing speed (mm/s)	20
Shear friction factor	0.12

**Table 3:** Chemical composition of SCM 420 (wt. %).

Cr	Mn	C	Ni	Ti	Cu	S	Si	P	Fe
1.20	0.90	0.18	0.18	0.08	0.22	0.02	0.26	0.023	Bal.

**Table 4:** Accuracy class of precision finished teeth.

Curve of profile deviation 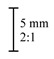 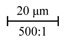	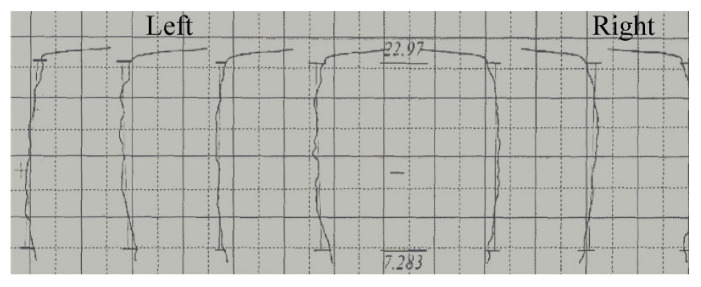
*F_α_* (ISO 1328)	17.2 μm (7 class)
Curve of helix deviation 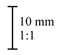 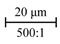	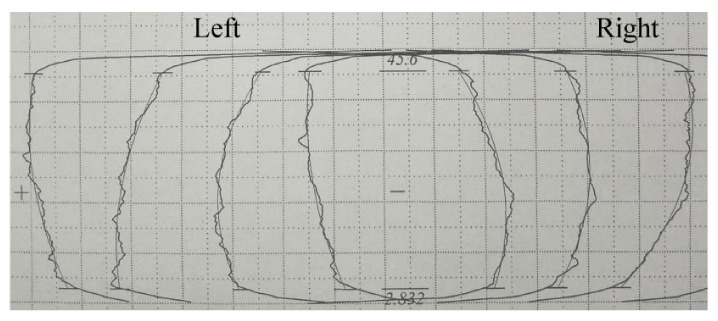
*F_β_* (ISO 1328)	21.1 μm (8 class)
*F_M_* (ISO 1328)	72.3 μm (100 μm)

## Data Availability

Not applicable.
